# From the archives: Inter-organelle communication, strategies to cope with ER stress, and a missing puzzle piece in light-dependent development

**DOI:** 10.1093/plcell/koae060

**Published:** 2024-02-20

**Authors:** Margot Raffeiner

**Affiliations:** Assistant Features Editor, The Plant Cell, American Society of Plant Biologists; Faculty of Biology & Biotechnology, Ruhr University Bochum, Bochum 44803, Germany

## May 2023: With a little help from my neighbor—highlighting the importance of inter-organelle communication

During the life cycle of seed plants, the transition from a vegetative to a reproductive stage is surely an incisive moment. This step includes the emergence of different floral organs, which is characterized by rapid changes in cell type and organ architecture, eventually involving dynamic changes in glycerolipid profiles (Nakamura 2015). Glycerolipids are the main component of biological membranes, and they are synthesized by phosphatidic acid (PA) phosphatases that dephosphorylate PA to produce diacylglycerol, a common precursor of major phospholipids. *Arabidopsis thaliana* (Arabidopsis) has 2 types of PA phosphatases: the soluble phosphatidate phosphohydrolases and the membrane-anchored lipid phosphate phosphatases (LPPs). Nguyen and Nakamura (2023) characterized 2 out of 9 Arabidopsis LPPs, LPPα2 and LPPε1 and investigated their role in glycerolipid metabolism (Fig.). They found that LPPα2 was localized to the endoplasmic reticulum (ER), a large organelle found in most eukaryotic organisms from yeast and animals to algae and plants. LPPε1, on the other hand, localized to the cytosolic side of the chloroplast outer envelope. Curiously though, despite this localization to different organelles, the authors showed that both enzymes act synergistically in ER phospholipid biosynthesis, highlighting the importance of inter-organelle communication. Here, the collaboration of the 2 distinctly localized proteins is ensured by the fact that the chloroplast outer envelope, the site of LPPε1 localization, is pushed into physical proximity of the ER membranes. The overexpression of LPPα2 and LPPε1 led to an enhanced production of only ER-localized but not chloroplastic glycerolipids that are thought to play a role in vegetative organs such as leaves. This affirms the importance of the characterized LPP isoforms in reproductive organ development.

**Figure.**
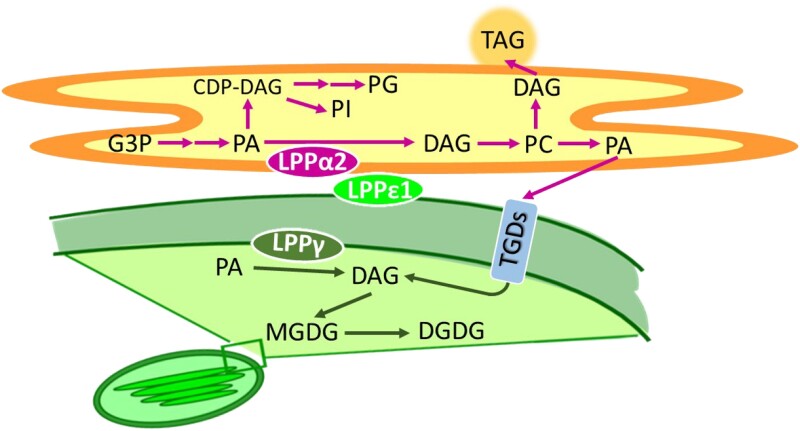
The ER-localized PA phosphatase LPPα2 and the chloroplast-localized LPPε1 catalyze ER glycerolipid metabolism in Arabidopsis. LPPε1, together with LPPα2, catalyzes only glycerolipid biosynthesis reactions taking place in the ER (orange, reactions indicated by arrows in magenta) but not glycerolipid biosynthesis pathways in the chloroplast (green, reactions indicated by green arrows). Adapted from Nguyen and Nakamura (2023), Figure 11.

## May 2019: ZIP the stress away—a Chlamydomonas bZIP transcription factor involved in ER stress regulation

The ER is not only a site for lipid biosynthesis but also for many other important events, such as protein synthesis, folding, and transport. Therefore, an accumulation of misfolded or unfolded proteins is a typical symptom of ER stress. This can be caused by a variety of biotic and abiotic factors, including pathogen infection or changes in temperature or salinity (Yamaoka et al. 2018). The unicellular microalga *Chlamydomonas reinhardtii* (Chlamydomonas) was shown to use the well-conserved ER stress sensor Inositol-requiring enzyme 1 (IRE1, *Cr*IRE1), a protein with both kinase and RNase activity, to monitor protein folding and eventually initiate the so-called unfolded protein response (UPR). The UPR counteracts ER stress and helps to restore regular protein metabolism. Yamaoka et al. (2019) investigated the events downstream of *Cr*IRE1-mediated ER stress sensing and identified the basic leucine zipper 1 (bZIP1) transcription factor (*Cr*bZIP1) as an essential player in Chlamydomonas ER stress management. The authors showed that *Cr*bZIP1 is spliced by *Cr*IRE1, leading to its translocation to the nucleus, where it exhibits transcription factor activity. Accordingly, Chlamydomonas *crbzip1* mutants grew poorly under ER stress conditions due to a failed induction of UPR. This includes a failure to induce the expression of UPR genes encoding for molecular chaperones, folding enzymes, or lipid and fatty acid biosynthetic enzymes. The latter is in line with the observation that *crbzip1* mutants were unable to produce the important glycerolipid diacylglyceryltrimethylhomoserine, as well as the fatty acid pinolenic acid, both involved in ER stress tolerance. Thus, in this study, Yamaoka et al. identified an important signaling cascade encompassing the ER stress sensor *Cr*IRE1, the transcription factor *Cr*bZIP1, and the target genes that catalyze the production of important compounds used to mitigate the stress. However, the authors also showed that this microalga uses an alternative ER stress-coping strategy in the absence of *Cr*bZIP1, namely the formation of lipid droplets, further confirming that lipid modifications are essential to its cell survival.

## May 1999: Finding a missing puzzle piece of the COP9 complex

Common to photosynthetic organisms, such as the green microalga *Chlamydomonas reinhardtii* (Chlamydomonas) or vascular plants like *Arabidopsis thaliana* (Arabidopsis), is their dependency on light. In plants, light is not only important for photosynthesis, where it is used as an energy source, but also for a developmental process termed photomorphogenesis. Here, light is perceived by photoreceptors, which then initiate a wide variety of downstream events, including the transition of the hypocotyl into the stem, the opening of the apical hook, or the expansion of the cotyledons and chloroplasts (Qin et al. 2020). In successful attempts in the early 1980s to identify the key components involved in this developmental process, genetic mutant screens revealed a set of *constitutively photomorphogenic* (*cop*), *de-etiolated* (*det*), and *fusca* (*fus*) mutants (*cop/det/fus*) in Arabidopsis. Subsequent and more extensive studies identified at least 11 pleiotropic COP/DET/FUS proteins that act as negative regulators of photomorphogenesis in darkness. Several of those proteins were shown to encode subunits of a large protein complex, initially termed the COP9 complex and later renamed to COP9 signalosome (CSN), which today is still considered the most important repressor of light-dependent development in Arabidopsis. However, it was also shown to be involved in other cellular and developmental processes in many different eukaryotes, including yeasts (e.g. *Saccharomyces cerevisiae*), *Drosophila*, or mammals. In 1999, Karniol et al. identified a novel component of the COP9 complex, the subunit FUSCA5 (FUS5). In lieu of a sequenced plant genome, Karniol et al. (1999) purified the COP9 complex from cauliflower, identified a 27-kD subunit, and then cloned the corresponding Arabidopsis cDNA based on the protein sequence. *fus5* mutants showed constitutive photomorphogenic phenotypes, indicating a disruption of the COP9 repressor complex activity in those mutants. The FUS5 protein was also shown to interact with other components of the COP9 complex, FUS6 and COP9, strengthening the idea of complex formation between the already known subunits and the newly identified one. Additionally, this study revealed phosphorylation events on FUS5 that may be of importance for the complex activity, because other of its components were previously shown to participate in kinase pathways. More recent studies further imply that, due to its structural characteristics, FUS5 (now also termed CSN7) may assist the assembly of the COP9 complex (Dessau et al. 2008).

## Dive Curated Terms

The following phenotypic, genotypic, and functional terms are of significance to the work described in this paper:

COP9 Gramene: AT4G14110
COP9 Araport: AT4G14110

FUS6 Gramene: AT3G61140
FUS6 Araport: AT3G61140

fus5 Gramene: AT1G02090
fus5 Araport: AT1G02090

LPP CHEBI: CHEBI:138807
